# Metagenomic Analysis of Microbial Contamination in the U.S. Portion of the Tijuana River Watershed

**DOI:** 10.3390/ijerph20010600

**Published:** 2022-12-29

**Authors:** Nicholas Allsing, Scott T. Kelley, Alexandra N. Fox, Karilyn E. Sant

**Affiliations:** 1 Department of Biology, San Diego State University, San Diego, CA 92182, USA; 2 Bioinformatics and Medical Informatics Program, San Diego State University, San Diego, CA 92182, USA; 3 School of Public Health, San Diego State University, San Diego, CA 92182, USA

**Keywords:** untargeted metagenomics, water, sanitation and hygiene (WaSH), Tijuana River and Estuary, impaired water bodies, stormwater flows, transborder water

## Abstract

The Tijuana River watershed is binational, flowing from Tijuana, Mexico into San Diego and Imperial Beach, USA. Aging sewage and stormwater infrastructure in Tijuana has not kept pace with population growth, causing overflows into this watershed during major rainfall or equipment failures. The public health consequences of this impaired watershed on the surrounding communities remain unknown. Here, we performed untargeted metagenomic sequencing to better characterize the sewage contamination in the Tijuana River, identifying potential pathogens and molecular indicators of antibiotic resistance in surface waters. In 2019–2020, water samples were collected within 48 h of major rainfall events at five transborder flow sites and at the mouth of the river in the US portion of the Tijuana River and estuary. After filtration, DNA was extracted and sequenced, and sequences were run through the Kaiju taxonomic classification program. A pathogen profile of the most abundant disease-causing microbes and viruses present in each of the samples was constructed, and specific markers of fecal contamination were identified and linked to each site. Results from diversity analysis between the sites showed clear distinction as well as similarities between sites and dates, and antibiotic-resistant genes were found at each site. This serves as a baseline characterization of microbial exposures to these local communities.

## 1. Introduction

Water, sanitation and hygiene (WaSH) is a global public health issue, stemming from substantial sources of municipal and industrial pollution and sewage contamination that have negatively impacted the health and overall quality of life of countless people. Nearly 2 billion people globally lack access to improved sanitation, resulting in an increased risk for communicable diseases [1,2]. Though over 97% of people in the United States have access to safe drinking water and sanitation infrastructure, only 43% of people in Mexico have these necessities [3]. This lack of safe drinking water especially affects developing countries, where diarrhea-related deaths in children younger than 5 years old are more common, with 1.7 billion cases of diarrhea being reported in this age group globally, and 9% of this age group’s 5.8 million deaths being attributed to this condition [4]. Additionally, cholera and typhoid fever are associated with a lack of clean drinking water, which count for an estimated 224,000 deaths globally [5,6]. In the U.S., according to a recent EPA report, more than half of the country’s water sources have a significant amount of pollution, with about 2.44% of community water systems and about 6% of Clean Water Act permittees not complying with the set standards of water quality [7,8]. Thus, there is a need for reliable methods for identifying and quantifying disease-causing microbes and viruses in highly contaminated waters.

The Tijuana River is an impaired water body located at the United States and Mexico border. This 1750 square mile watershed crosses the U.S. border, with most of its area located in Mexico [9]. Flowing south to north, it drains into the Tijuana River Estuary which then deposits into the Pacific Ocean. Following rapid urbanization in Tijuana and insufficient upgrades to infrastructure, the water quality of the Tijuana River has been degrading for decades. This degradation leads to sewage pollution in both the United States and Mexico which may pose a public health risk to those living along the border [10]. The lower six miles of the Tijuana River and the Tijuana River Estuary which are jointly called the Tijuana River Valley are classified as impaired water bodies according to the U.S. Clean Water Act due to unacceptable quantities of heavy metals, bacteria, sediment, and trash existing in the water [10].

Measures by both countries to treat the water at the border have been inadequate, with beach closures becoming increasingly common and water quality frequently failing to meet standards [11]. The Comisión Estatal de Servicios Públicos de Tijuana (CESPT) manages the operations of several water treatment facilities in Tijuana and at the international border in San Diego County, but costly upgrades have been outpaced by rapid urbanization. Though the majority (estimated at 80–85%) of Tijuana households have proper sewer connections, this means that several hundred thousand dwellings may not be properly connected to the grid, generating sewage and waste that may be carried into the Tijuana River watershed through surface and groundwater contamination.

The U.S. border communities of San Ysidro (the southernmost part of the City of San Diego) and Imperial Beach are located directly adjacent to the U.S.–Mexico border and within the boundaries of the Tijuana River. These are both characterized as socioeconomically disadvantaged communities, and overall have an elevated risk of chronic diseases compared to surrounding cities in San Diego county [12]. One potential factor of this increased risk of disease is the contamination present in the water of the Tijuana River, and numerous programs are underdevelopment or underway to improve water and air quality at the U.S.–Mexico border. However, these current environmental health challenges, such as microbial and chemical loading into these coastal waters, are persistent, and may have lasting effects that are difficult to mitigate.

Identifying the disease-causing microbes and viruses present in the water will allow us to better understand the effects that sewage contamination has on public health and aid in the creation of a targeted solution. Current methods used to identify disease-causing microbes and viruses in environmental water samples based on PCR and culturing tend to be too narrowly focused, only assessing a specific species or genus of bacteria. Furthermore, while new approaches to detect fecal contamination, such as the utilization of HF183 and crAssphage primers that target human fecal-associated bacteria and phages, have presented more options for detecting incidences and levels of fecal contamination, identification of specific potential pathogens still needs to be completed separately from the detection of sewage contamination. These methods also do not provide information about antibiotic resistance, which can influence pathogenesis and persistence. Due to these limitations, there are many disease-causing microbes and viruses that go undetected, posing a serious health risk to affected communities.

Here, we apply an untargeted metagenomic approach to detect sewage contamination and simultaneously identify and verify the presence of disease-causing microbes, viruses, and antibiotic resistance genes in samples collected from the U.S. portion of Tijuana River and Estuary. By using metagenomic sequencing to determine both microbial and viral diversity across multiple sites along the Tijuana River, we were able to identify multiple independent indicators to monitor fecal contamination levels. In addition, we applied a novel bioinformatics approach that calculated the breadth-of-coverage (BOC) to selected reference genomes to verify rapid metagenomic identification. Together, these approaches have exciting future applications for rapidly monitoring water systems and could be used to help address the challenges of water contamination, not just for the communities near the Tijuana River, but for those impacted across the globe.

## 2. Materials and Methods

### 2.1. Sample Collection, E. coli and Coliform Measurements

**Figure 1:**
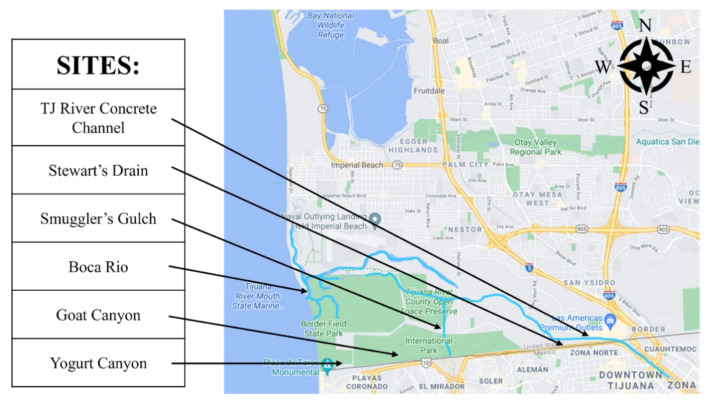
Location of sampling sites, November 2019–February 2020.

Twenty-two water samples were taken from six sites on 22 November 2019, 6 December 2019, 12 February 2020, and 24 February 2020. Figure 1 identifies the locations of the TJ River Concrete Channel, Stewart’s Drain, Smuggler’s Gulch, Boca Rio, Goat Canyon, and Yogurt Canyon sites. Coordinates are provided in Table S1 and assigned sample numbers are provided in Table S2. All sites, except Boca Rio, are located at the U.S.–Mexico border and receive transboundary stormwater flows. Boca Rio is an estuarine site with tidal flows at the mouth of the Tijuana River as it enters the Pacific Ocean. The TJ River Concrete Channel, Stewart’s Drain, and Smuggler’s Gulch sites are concrete-lined basins or channels, and the Goat Canyon and Yogurt Canyon sites are natural basins. Samples were collected >24 h post rainfall, which maximized stormwater input while minimizing safety risk due to flooding. For two dates, samples could not be collected at the Goat Canyon site due to low water depth at the time of sampling. Grab samples were collected from each site in sterile Whirl-Pak^®^ bags, transported to the lab in coolers with ice packs, and immediately filtered through 0.22 μm Sterivex^®^ filters until clogging prevented filtration. Filtration volumes are provided in Table S2.

### 2.2. DNA Extraction, Purification, and Sequencing

DNA was extracted and purified from the samples using the Qiagen DNeasy^®^ PowerWater^®^ Sterivex^®^ kit according to the manufacturer’s instructions. Concentration and purity of the extracted DNA was then measured using a NanoDrop^®^ Spectrophotometer. Library preparation was completed using the Nextera XT DNA Library Preparation Kit. DNA sequencing was completed by the Microbial Genome Sequencing Center (MiGS) at an approximate read depth of 25 M using the NextSeq 200 platform, which returned sequence files with forward and reverse reads.

### 2.3. Processing and Taxonomic Classification

The sequences were quality controlled using fastp, an “ultra-fast all-in-one FASTQ preprocessing” program [13]. Filtering was based on length, number of unknown bases, and a Phred quality score (Q) cutoff of 15 to exclude any bases with a base call accuracy lower than 96.8%. Throughout the experiment, the program GNU Parallel was used to execute commands simultaneously, taking advantage of multi-threading [14]. Kaiju, a metagenomic taxonomic classification program [15], was used to identify matches to the NCBI BLAST non-redundant protein database (nr_euk). To align the samples to the protein database of nr_euk, which includes Bacteria, Eukaryotes, Archaea, and Viruses, Kaiju translated each DNA sequence to a protein sequence before finding matches of a minimum length of 11 and a minimum match score of 65, allowing for 3 mismatches, and counting alignment hits. The output from Kaiju was then converted into a readable table using the included kaiju2table program, listing the domain, kingdom, phylum, class, order, family, genus, and species of each positive hit.

### 2.4. OTU Table Creation and Modification

The output files of the kaiju2table program were combined and converted into a single metagenomic Operational Taxonomic Unit (OTU) table using a Python script, listing the number of hits for each OTU and organizing the data by sample [16]. The taxonomic ranks from domain to genus were removed and hits that did not have a species-specific match were renamed accordingly as either unclassified, where the reads were not matched at all, or could not be assigned to a (non-viral) species, where the hit was assigned to a taxonomic level above species.

### 2.5. Disease-Causing Microbe and Virus Identification

Consulting the 2015–2019 County of San Diego Health and Human Services Agency’s Reportable Diseases and Conditions by Year [17], a manual search was conducted of disease-causing Bacteria, Eukaryotes, and Viruses present in the Kaiju results. The number of hits for each species that matched San Diego County’s list was counted then averaged across the samples.

### 2.6. Breadth of Coverage

Reference genomes for *Arcobacter cryaerophilus*, *Escherichia coli*, *Human immunodeficiency virus 1*, and seven different *Salmonella enterica* sub-species were obtained from NCBI Assembly. Indices for the reference genomes were built using bowtie2-build [18]. The sequence reads were then mapped to the reference genomes using bowtie2 [19,20], creating .sam files. The .sam files were then converted to .bam files and were sorted and indexed. Following the concept laid out by Matthias Scholz in “SAMtools: get breadth of coverage”, the percent BOC was then calculated by dividing the number of bases covered per sample per reference genome by the total length of the reference genome.

### 2.7. Diversity Analyses

The modified metagenomic OTU table without viral species and a mapping file with sample metadata were loaded into R to perform diversity analysis. The tables underwent zero replacement using the zCompositions package [21], converting all zero values to pseudo-counts with Bayesian-multiplicative replacement. The centered-log ratio (clr) was then used to transform the data, comparing the abundance of each species to the geometric mean of the sample [22,23]. Non-metric multidimensional scaling (NMDS) was used with a Euclidean distance matrix to calculate and map similarities and differences between samples on a two-dimensional plane. Several beta-diversity analyses were then completed including analysis of variance, multivariate homogeneity of group dispersions, and Tukey’s test [24,25].

To assess alpha diversity, the modified metagenomic OTU table generated from Kaiju was also imported into QIIME 2 as a feature table and converted into a .qza file for further analysis [26]. Using QIIME 2, several forms of alpha diversity metrics were performed, including a quantification of the number of observed features and the calculation of Shannon Entropy and Pielou Evenness. These metrics were then output and merged into one .tsv file, and the corresponding metadata were added. This alpha diversity metric file was then imported into R to generate plots for the relevant alpha diversity statistics.

### 2.8. Fecal Contamination Analysis

A list of bacteria commonly associated with the fecal contamination PCR primer HF183, based on J P Nshimyimana et al. [27], was compiled from the Kaiju results OTU table. These bacteria included *Bacteroides vulgatus*, *Bacteroides uniformis*, *Bacteroides fragilis*, *Bacteroides stercoris*, *Bacteroides dorei*, *Prevotella ruminicola*, *Bacteroides stercoris* CAG:120, *Bacteroides intestinalis* CAG:315, *Bacteroides uniformis* CAG:3, *Bacteroides intestinalis* CAG:564, *Bacteroides fragilis* CAG:558, *Bacteroides vulgatus* CAG:6, *Bacteroides dorei* CAG:222, and *Bacteroides fragilis* CAG:47. Another list with common human-associated crAssphages was also created to isolate the common human fecal inhabiting phage from the metagenomic data [28,29,30,31,32], curated from the NCBI Taxonomy database [33]. These include uncultured crAssphage, crAssphage LMMB, crAssphage sp. C0521BD4, crAssphage cr7_1, crAssphage cr85_1, and crAssphage cr8_1. The sums of the HF183 bacteria and the crAssphages were appended to the data, replacing the individual species counts before undergoing clr transformation. The HF183 and crAssphage data were isolated from the transformed OTU table and underwent several forms of statistical analysis for correlation including analysis of variance (ANOVA), Spearman correlation, Kendall correlation, Pearson correlation, and regression analysis.

Total coliform and *E. coli* measurements were performed using the IDEXX Colilert test. Samples were diluted based upon historical records of coliform and *E. coli* concentrations in the Tijuana River, typically by 10,000–1,000,000X. Most probable numbers (MPNs) were calculated following manufacturer instructions. Total coliform and *E. coli* MPNs are provided by site and date in Table S3. No correlations were noted with precipitation or transboundary flow gauge readings (R^2^ < 0.3) [34].

### 2.9. Antibiotic Resistance Genes (ARGs)

Raw sequencing reads from each site were converted from .fastq format into .fasta with the program seqtk [35]. Two groups of antibiotic resistance genes (ARGs), antibiotic inactivation and antibiotic efflux, were then downloaded from the Comprehensive Antibiotic Resistance Database (CARD) [36] using the qualifiers part_of, is_a, participates_in, has_part, and nucleotide. A BLAST database was then created for each antibiotic resistance group. Using blastn [37] with 100% identity and 100% query coverage filters, the raw reads were aligned to the database. The blastn results were then organized into a table with information on the alignment length, mismatches, gaps, start and stop locations of the query and subject, and e-value. The number of matches to a specific antibiotic resistance gene for each sample was calculated and combined into a separate table, similar to an OTU table. The relative abundance of each gene for each sample was calculated by dividing by the number of reads in each sample’s sequencing files. This ARG table was imported into R and the five most common genes by average relative abundance were graphed and comparisons by site were calculated using Dunn’s test [38]. The sum of the relative abundances for each site was also graphed and *p*-Values were calculated using Dunn’s test.

### 2.10. Scripts and Data

All scripts that were used for the experiments can be found at https://github.com/nickallsing/TJ_River_Project (accessed on 1 November 2022) as well as sample input and output data from this experiment. Scripts for the assessment of antibiotic resistance gene markers can be found at https://github.com/nickallsing/Find_ARG (accessed on 1 November 2022). The sequences that were used can be found under the project accession number PRJEB57859.

## 3. Results

### 3.1. DNA Concentration and Sequence Quality

The DNA and sequences were at a normal concentration and were high-quality reads. Results from the NanoDrop showed a range of 3.4 to 11.7 ng/μL for all samples. After performing quality control on the sequences, an average of 99.24% of the reads passed the set filters checking for quality, read length, and missing bases. An average GC content of 47.59% and 47.54% were reported before and after filtering, respectively. The fastp quality control program also reported an average duplication rate of 19.86% across samples.

### 3.2. Metagenomic Results with Disease-Causing Microbes and Viruses

Metagenomic analysis revealed the most common hits present in the samples, the ten highest averages of which are shown in Table 1. The most common species, on average, in all 22 samples was the bacteria *Arcobacter cryaerophilus*, with 140,491.1 average hits. *A. cryaerophilus* is a diarrheal pathogen of emerging interest, and thus is not commonly surveilled by health systems. The names of several disease-causing bacteria, eukaryotes, and even some viruses listed in the San Diego County Reportable Diseases and Conditions by Year: 2015–2019 were found in the Kaiju metagenomic OTU table. Table 2 lists the 11 most common disease-causing bacteria, eukaryotes, and viruses found as well as each species’ average number of hits across all 22 samples. *Salmonella enterica*, *Vibrio parahaemolyticus*, and *Streptococcus pneumoniae* were the three most common bacteria, with 1890.4, 1602.4, and 1009.0 average hits, respectively. The most commonly identified eukaryote was the parasite *Trichomonas vaginalis*, and HIV-1 was the most common disease-causing virus, with 14.2 hits on average.

### 3.3. Breadth of Coverage

The samples from the transborder Stewart’s Drain site at each sampling date were chosen to test the BOC verification method due to their high relative abundance of Salmonella enterica, *Escherichia coli*, *Arcobacter cryaerophilus*, and HIV-1. Table 3 shows the results of the percent BOC for these genomes across the samples collected at Stewart’s Drain (Samples 1, 8, 14, and 20), which had the highest *E. coli* and total coliform concentrations (Table S3). This site was selected because of its close proximity to the international wastewater treatment plant and pump station, which had failed several times during the study period corresponding with transborder spills (including 11/18/19) and was hypothesized to contribute to higher levels of fecal indicator bacteria in Stewart’s Drain. HIV-1 BOC was approximately 0%, suggesting that the detection of HIV-1 in these samples could be a false positive. This demonstrates the importance of BOC analysis in the validation of metagenomic sequencing.

### 3.4. Diversity Analysis

**Figure 2:**
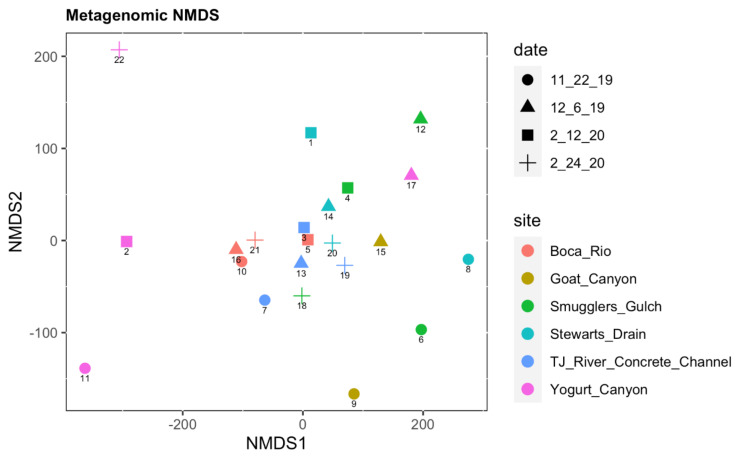
Metagenomic Non-Metric Multidimensional Scaling Plot.

After transforming the data using the centered-log ratio and performing NMDS, the coordinates displayed a pattern in which the Yogurt Canyon samples were clearly distinct from the other sites, but also from other Yogurt Canyon samples. Yogurt Canyon samples 2, 11, and 22 were each at a far end of the plot, away from the group and away from each other, as shown in Figure 2. The most closely grouped site was Boca Rio, with Samples 10, 16, and 21 very close and Sample 5 also not clearly separated. Permutational analysis of variance (PERMANOVA) between dates showed no statistical significance (*p* = 0.4275), while variance between sites was significant (*p* = 0.0016). Graphed values using Tukey’s test for significant differences showed the distance to centroid between sites and dates, with a similar trend from ANOVA, with sites having a greater variance than dates (Figure S1).

### 3.5. Alpha Diversity

**Figure 3:**
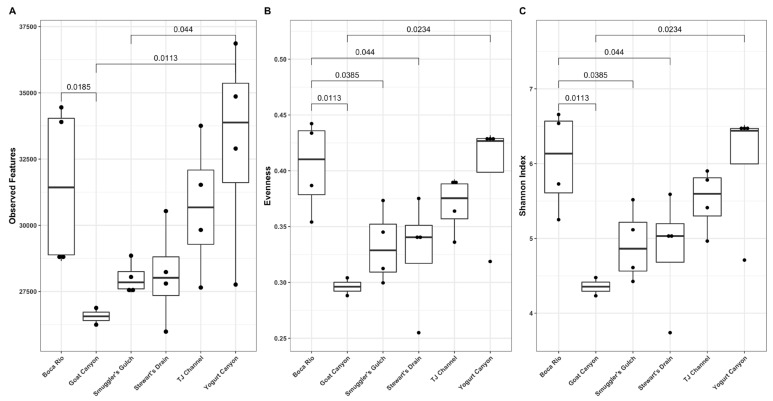
Alpha diversity metrics across sites based on observed features (**A**), evenness (**B**), and Shannon Index (**C**).

Significant differences in alpha diversity were observed in the number of features, evenness, and Shannon index between sites along the Tijuana River (Figure 3). The number of observed features was significantly higher at the Yogurt Canyon site compared to Goat Canyon and Smuggler’s Gulch with a Dunn’s test *p*-value of 0.0454 and 0.0337, respectively. Evenness metrics showed significant differences between Boca Rio and the Goat Canyon, Smuggler’s Gulch, and Stewart’s Drain sites with respective *p*-values of 0.0113, 0.0385, and 0.044. Yogurt Canyon also varied in this metric from the Goat Canyon site with a *p*-values of 0.0234. Lastly, Shannon Indexing revealed the same relationships between the sites that were present in the Evenness results.

### 3.6. Fecal Contamination Analysis

**Figure 4:**
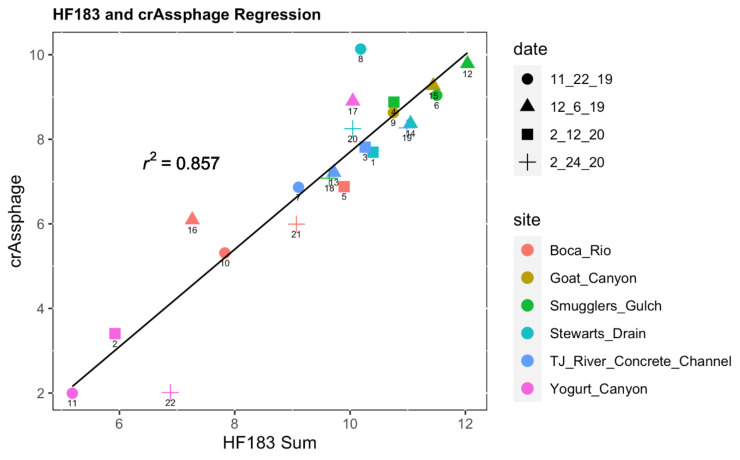
Regression Plot of crAssphage and HF183 Bacteria.

Coliform and *E. coli* most probable number (MPN) tests showed a high level of fecal contamination, especially in Samples 17 (Yogurt Canyon, 12/6/19) and 20 (Stewart’s Drain, 2/24/20), with several other samples reaching the maximum measurements (Table S3). Coliform and *E. coli* levels were the lowest in the Tijuana River Estuary (Samples 11, 2, 22, 21, 10, and 16), shown in Table S3. After isolation of HF183 bacteria and crAssphage from metagenomic data, correlation analysis showed a very significant relationship between the two groups (*p* < 3.143 × 10^−6^ for all methods; Table S4), with an R-squared value of 0.857, shown in Figure 4.

### 3.7. Antibiotic Resistance Genes (ARGs)

The five inactivation-based ARGs that were present the most on average were a beta-lactamase resistance gene *oxa2* plasmid R46 integron (NCBI Accession: M95287.4), a streptomycin resistance aadA (Ant(3″)-IIa) gene from *E. coli* plasmid R538-1 (NCBI Accession: X02340.1), an erythromycin resistance gene *mph* from an uncultured bacterial plasmid (NCBI Accession: DQ839391.1), a beta-lactamase 2-like protin *bla-OXA2* from *Stenotrophomonas maltophila* (NCBI Accession: KJ138219.1), and another streptomycin resistance *aadA* gene from *E. coli* (NCBI Accession: AF550679.1). The five efflux-based ARGs that were present the most on average were a tetracycline resistance gene *tetR(39)* from *Acinetobacter sp.* LUH5605 (NCBI Accession: AY743590.1), a tetracycline resistance structural protein *tetA* from *Acinetobacter* sp. LUH5605 (NCBI Accession: AY043299.1), a multi-drug resistant protein *qacEdelta1* from *Pseudomonas aeruginosa* (NCBI Accession: U49101.1), a chloramphenicol resistance protein *cmlA5* from uncultured bacterium plasmid pSp1 (NCBI Accession: AY115475.1), and a chloramphenicol-resistance protein *cmlA* from *Pseudomonas aeruginosa* (NCBI Accession: M64556.1).

**Figure 5:**
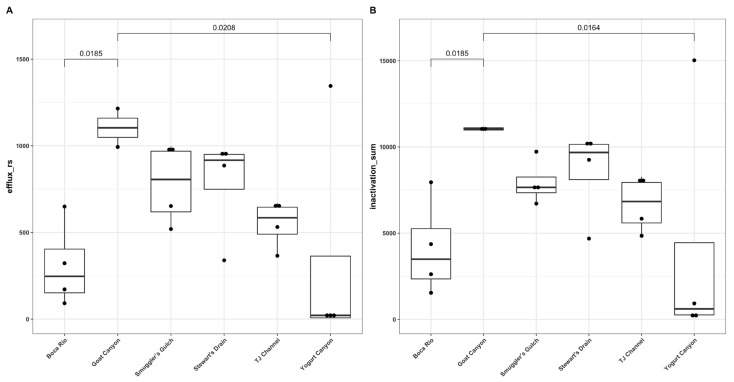
Sum of efflux (**A**) and inactivation (**B**) genes found in water samples, by location.

The relative abundances of *oxa2* and *bla-OXA2* genes were significantly different between Stewart’s Drain and the Boca Rio, TJ River Concrete Channel, and Yogurt Canyon with *p*-values of 0.0143, 0.0385, and 0.009, respectively. The *oxa2* and *bla-OXA2* genes were significantly different within the same sites as with *p*-values of 0.0143, 0.0256, and 0.0076. The only significant difference with the *aadA* genes occurred between Goat Canyon and Yogurt Canyon (*p* = 0.0454). There were no significant differences between sites in the *aadA* (ANT(3″)-IIa) and *mph* inactivation genes. In the efflux ARGs, *tetA* and *qacEdelta1* were significantly different between the Stewart’s Drain and Yogurt Canyon sites (*p* = 0.0192 and *p* = 0.0294, respectively). Efflux gene *cmlA5* abundances were different between Goat Canyon and the Boca Rio and Yogurt Canyon site (*p* = 0.0329 and *p* = 0.0145, respectively). The only significant difference in the *cmlA* gene was between Goat Canyon and Yogurt Canyon (*p* = 0.0262). There were no significant differences found among sites with the *tetR* gene. When the relative abundances of each group of genes were summed, both the inactivation and efflux genes showed significant differences between Goat Canyon and the Boca Rio and Yogurt Canyon sites (Figure 5). In both groups, the abundance of Goat Canyon was higher than that of Boca Rio and Yogurt Canyon.

## 4. Discussion

Metagenomic techniques have the potential to be a powerful public health tool for tracking current and emerging pathogens. In this study, we demonstrate the utility of untargeted metagenomic sequencing for analyzing contaminated surface waters as an epidemiological indicator and diagnostic tool for water quality investigations. We examined transborder stormwater flows from Tijuana, Mexico into San Ysidro and Imperial Beach, California during the 2019–2020 winter season. The information obtained from these experiments provided considerable insight into these highly contaminated areas including estimates of their relative microbial diversity, the types of microbes present with verification, evidence of significant antimicrobial resistance markers, and levels of fecal contamination. These insights will be useful for identifying potential future epidemiological challenges in the surrounding areas.

Diversity analysis showed the clear compositional separation of some sites, allowing us to better understand how diversity of species changes based on location. When comparing differences in alpha and beta diversity based on date, there was no significant trend; however, differences based on site were significant (Figure 3 and Figure S1), with the distinction of Yogurt Canyon and Boca Rio sites from the other samples bring particularly noteworthy. This difference can be partially explained by the location and hydrology of these two sites. Both Boca Rio and Yogurt Canyon, as they are estuarine, experience tidal influence. At these sites, especially at Boca Rio, the contamination that may have entered the river upstream is diluted by the ocean water. Yogurt Canyon also experiences this ocean mixing due to its location in the coastal floodplain and tidal flows daily. Our data suggest that this tidal influence significantly impacts the microbial and viral makeup of the environment, while causing significant changes to microbial ecology on a site-to-site basis.

Fecal analysis showed a strong correlation between HF183-associated bacteria and crAssphage (Figure 4) and allowed us to clearly determine differences in the levels of fecal contamination at different sites. These data were corroborated with total coliform and *E. coli* measurements done at the same sites (Table S3). Yogurt Canyon sites 2, 11, and 22 were closely grouped on the regression plot at the lowest level of HF183 bacteria and crAssphage. These same sites also had the lowest MPN of coliforms and *E. coli*, demonstrating the effectiveness of this test. Similarly, Boca Rio sites 10, 16, and 21 were also low in metagenomic fecal levels and in coliform and *E. coli* MPN data. These data show that Yogurt Canyon, except for site 17, and Boca Rio have a much lower amount of fecal contamination, likely due to their proximity to the ocean where dilution can occur. Interestingly, site 8, had elevated crAssphage levels compared to HF-183. This sample was collected within 3 days of transborder “sudden flow from Mexico” reported to the California Water Boards, which may explain some of the deviation in BOC and crAssphage:HF-183 compared to other samples. While regulatory agencies consider the best methods to predict fecal contamination in surface waters, these data show strong correlations between two fecal indicators, HF183 (bacterial) and crAssphage (viral). Both fecal indicators are considered to be highly human-specific, and neither requires measurement of live microbes which allows those collecting samples more flexibility in the timing of laboratory analyses. However, future studies can focus more closely on the relationships between metagenomic fecal contamination analysis and more traditional methods to understand factors that predict the live or potentially infectious fraction of those measured using metagenomics.

Identifying the exact pathogens that may be present in environmental samples allows for the targeted treatment of contamination, as one pathogenic species may require a different solution than another. In our samples, presence of *Salmonella enterica*, *Escherichia coli*, *Arcobacter cryaerophilus*, and *Trichomonas vaginalis* within the metagenomic data demonstrated the ability to identify common disease-causing agents. *Salmonella enterica* was the one of the most commonly observed pathogenic bacteria in our samples, which is widely associated with Salmonellosis and diarrhea. *Arcobacter cryaerophilus*, an emerging intestinal pathogen associated with acute diarrhea [39,40], was not included in the San Diego County List of Reportable Diseases but was one of the most common bacterial species found in the data. Because of the high breadth of coverage (79–95% for all samples), it is likely that this is a true finding of *A. cryaerophilus*—a pathogen that is not commonly reported nor surveilled by health systems. This demonstrates the power of untargeted metagenomic sequencing as a method to discover pathogens that may not have initially been considered.

One of the drawbacks of metagenomic analysis is the possibility of obtaining a false positive result. There are many different ideologies when it comes to identifying and validating the results found in metagenomic data, such as clinical laboratory validation and specific gene identification [41,42]. To validate our metagenomic data and remove any clear false positives, we utilized the BOC test. Our use of this strategy was novel, increasing our data rigor and reproducibility beyond typical reporting standards. Samples from Stewart’s Drain, a transborder flow site where contamination commonly occurs, at each sampling date were chosen to monitor the changes in BOC at a single site. These samples were also chosen due to their high metagenomic hit level and had ranges of 1.85–10.14% coverage for *Salmonella enterica* species, 11.87–78.56% for *E. coli*, 79.60–94.81% for *Arcobacter cryaerophilus*, and 0% for HIV-1, allowing us to validate the metagenomic results, declaring the presence of HIV-1 as most likely a false positive. While there is at present no standard minimum percent BOC for confirming the presence of a certain microbe or virus, we were able to clearly rule out negative results, such as HIV-1. Moreover, the low BOC for the *Salmonella* genomes indicates uncertainty in terms of which species or strain of Salmonella was present in the samples. Important future goals of this research will be to determine a percent BOC threshold that can be used to confidently validate metagenomic results and apply metagenomic and scaffolding tools to aid in the verification process and determine novel species and strains.

Drug resistance is frequently conferred through several key mechanisms, including efflux function and lactamase function. Increased efflux activity allows for rapid and effective export of toxicants, decreasing microbial concentrations [43,44,45]. Beta lactamases are enzymes that confer multidrug resistance through hydrolysis of the lactam antimicrobial enzymes [46]. In doing so, they render the actions of many common antibiotics (including penicillins and cephalosporins) ineffective. In our study, beta lactamases were significantly elevated, especially in sites nearest sewage spills and heavy transborder stormwater flows. Efflux genes followed this same spatial and quantitative pattern. These data suggest that microbes with conferred antibiotic resistance are entering the Tijuana River and Estuary through these spills and stormwater flows. Though it is not uncommon to detect ARGs in sewage, the abundance of ARGs in our samples may indicate increased use of antibiotics in Tijuana. However, the flow of these ARGs into the Tijuana River and coastal waters is a potential concern. Though beach closures occur during and for several days following major rainfall events, it is possible that the persistence of these genes and their carriers in the environment may have implications for human health, local microbial gene flow and drift, and overall ecosystem health.

Wastewater epidemiology is a modern tool to assess communicable diseases in local populations, as recently deployed around the world to assess prevalence of the SARS-CoV-2 virus [47], including in the Tijuana River [48]. While the present study results have some interesting implications, there are several limitations and important factors to consider in the application of this approach to public health questions. As with nearly all molecular methods to survey microbes, we do not know with certainty if the microbes found by the search were alive and present at the minimum infectious concentration required to cause disease. Therefore, detection of these pathogens in these waters does not suggest that the diseases themselves are transmissible through the surface water. However, any live and transmittable microbes and viruses may influence disease through normal activities such as swimming, surfing, and eating locally caught fish. For example, previous publications have demonstrated metagenomic water quality monitoring to reveal information about associated disease. Researchers have shown that sequences from environmental water samples in Haiti and their metagenomic analysis allowed for the microbial characterization of specific sites, which revealed low numbers of the harmful O1 and O139 strains of *V. cholerae*, which agreed with the decline of the occurrence of the disease in the area [49]. Therefore, use of these metagenomics strategies can inform those in public health about emerging outbreaks or underdiagnosed and under resourced conditions within the local population. Given this information, we believe that the results obtained in this study can also provide relevant and helpful information to the public and those who can address the causes of environmental contamination.

## 5. Conclusions

Overall, these experiments have exciting applications for detecting potential disease-causing microbes and viruses present in environmental water samples, verifying these results using reference genome sequence alignment, comparing microbial and viral diversity across sites, identifying antibiotic resistance, and measuring human fecal contamination levels all with the same data. There was a clear relationship between proximity to the transborder sites (versus estuarine) and fecal contamination, and the genomes of pathogenic species were detected at these fecal contaminated sites. Though it is very rare to have direct contact with the surface waters in these transboundary flows, we have shown that these species persist out to the mouth of the river and therefore recreational exposures (surfing, swimming, hiking) are possible in the local communities. We are optimistic about the use of untargeted metagenomic sequencing to fight the increasingly important and international problem of sewage contamination and pathogen detection in community surface waters.

## Figures and Tables

**Table 1:** List of top metagenomic results.

Species	Average Number of Hits *
*Arcobacter cryaerophilus*	140,491.1
*Pseudoarcobacter acticola*	98,044.3
*Simplicispira metamorpha*	76,102.6
*Bacteroides graminisolvens*	59,197.3
*Tolumonas auensis*	57,818.1
*Arcobacter suis*	50,397.2
*Acinetobacter johnsonii*	39,478.1
*Aeromonas media*	36,381.4
*Arcobacter butzleri*	32,238.7
*Rheinheimera sp. LHK132*	32,046.6

* Average number of matches across all 22 metagenome libraries.

**Table 2:** List of Disease-Causing Bacteria, Eukaryotes, and Viruses.

Bacterial Species	Average Hits *	Eukaryotic Species	Average Hits
*Salmonella enterica*	1890.4	*Trichomonas vaginalis*	97.8
*Vibrio parahaemolyticus*	1602.4	*Plasmodium ovale*	36.2
*Streptococcus pneumoniae*	1009.0	*Plasmodium vivax*	29.1
*Vibrio alginolyticus*	462.8	*Cyclospora cayetanensis*	25.2
*Bordetella pertussis*	459.6	*Plasmodium falciparum*	23.8
*Francisella tularensis*	360.3	*Plasmodium yoelii*	21.7
*Vibrio vulnificus*	340.5	*Entamoeba histolytica*	18.4
*Neisseria meningitidis*	312.0	**Viruses**	**Average Hits**
*Yersinia enterocolitica*	273.5	*HIV-1*	14.2
*Mycobacterium tuberculosis*	268.0	*Hepatitis C*	6.1
*Listeria monocytogenes*	101.0	*Hepatitis B*	3.7

* Average number of matches across all 22 metagenome libraries.

**Table 3:** Percent Breadth of Coverage on Stewart’s Drain Samples.

Species	Sample 1 2/12/20	Sample 8 11/22/19	Sample 14 12/6/19	Sample 20 2/24/20
*S. enterica* 45157	2.12%	9.77%	6.21%	3.48%
*S. enterica* DA34827	2.62%	10.09%	6.77%	3.31%
*S. enterica* FDAARGOS 94	2.50%	10.14%	6.71%	3.72%
*S. enterica* FDAARGOS 878	2.47%	10.00%	6.59%	3.63%
*S. enterica* LT2	2.48%	10.01%	6.63%	3.66%
*S. enterica* SA20021456	1.85%	8.06%	5.27%	2.88%
*S. enterica* SA20100201	1.97%	8.79%	5.68%	3.15%
*Escherichia coli*	11.87%	78.56%	55.51%	26.03%
*Arcobacter cryaerophilus*	91.27%	79.60%	94.81%	94.20%
HIV-1	0%	0%	0%	0%

## Data Availability

The metagenomics sequencing data presented in this study are openly available in the European Nucleotide Archive (ENA), project accession number PRJEB57859.

## Supplementary Materials

The following supporting information can be downloaded at: https://www.mdpi.com/article/10.3390/ijerph20010600/s1, Figure S1: Beta-Diversity Analysis Across Sites (A) and Dates (B); Figure S2: Genes conferring antibiotic resistance through efflux mechanisms, found at each site in the Tijuana River and Estuary; Figure S3: Genes conferring antibiotic resistance through inactivation (including beta-lactamase) mechanisms, found at each site in the Tijuana River and Estuary; Table S1. Site names and coordinates; Table S2. Summary table of Total Coliform and *E. coli* counts (MPN/100 mL) at sampling sites between October 2019 and February 2020; Table S3. Sample filtration volumes; Table S4. Coliform and *E. coli* results; Table S5. HF183 and crAssphage correlation results.
