# Supplementary material for: Metagenomic Analysis of Microbial Contamination in the U.S. Portion of the Tijuana River Watershed
Source: Int J Environ Res Public Health. 2022 Dec 29;20(1):600. doi: 10.3390/ijerph20010600 (PMC9819409; doi:10.3390/ijerph20010600)
Supplement: Supplementary file 1 [file ijerph-20-00600-s001.zip › ijerph-2098052-supplementary.pdf]

SUPPLEMENTAL TABLES AND FIGURES

Metagenomic Analysis of Microbial Contamination in the U.S. Portion of the Tijuana River Watershed

Nicholas Allsing, Scott Kelley, Alexandra Fox, and Karilyn E. Sant

Supplemental Data: Table of Contents

Figure S1: Beta-Diversity Analysis Across Sites (A) and Dates (B)

Figure S2: Genes conferring antibiotic resistance through efflux mechanisms, found at each site in the Tijuana River and Estuary.

Figure S3: Genes conferring antibiotic resistance through inactivation (including beta-lactamase) mechanisms, found at each site in the Tijuana River and Estuary.

Table S1. Site names and coordinates.

Table S2. Sample filtration volumes

Table S3. Summary table of Total Coliform and E. coli counts (MPN/100ml) at sampling sites between October 2019 and February 2020.

Table S4. HF183 and crAssphage Correlation Results

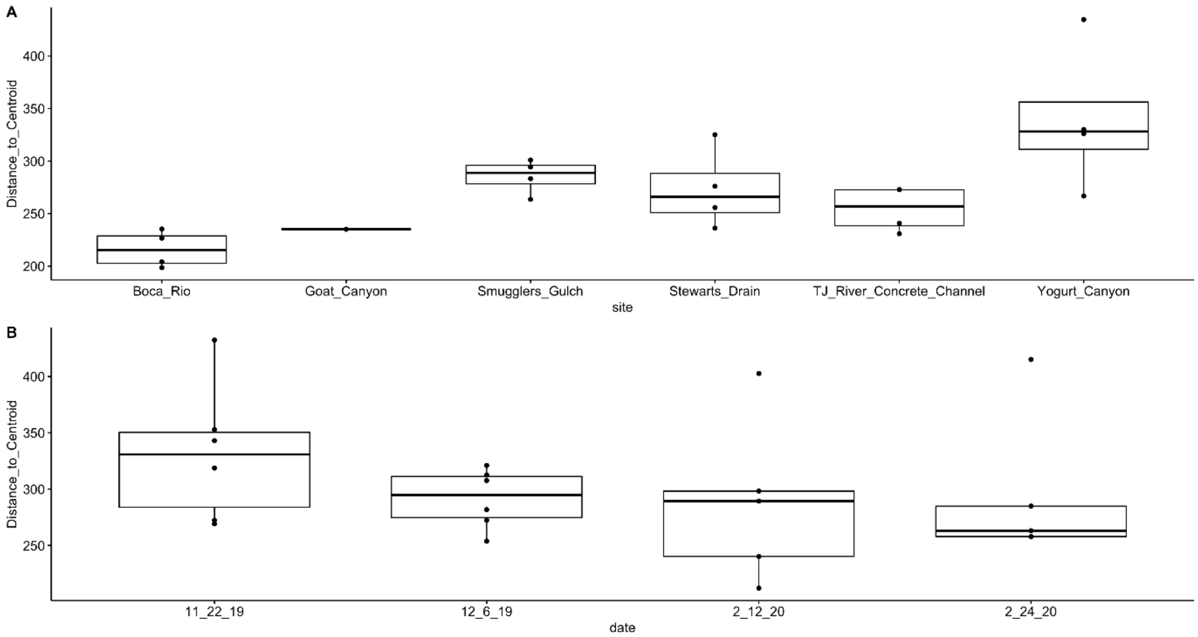

Figure S1. Beta-Diversity Analysis Across Sites (A) and Dates (B)

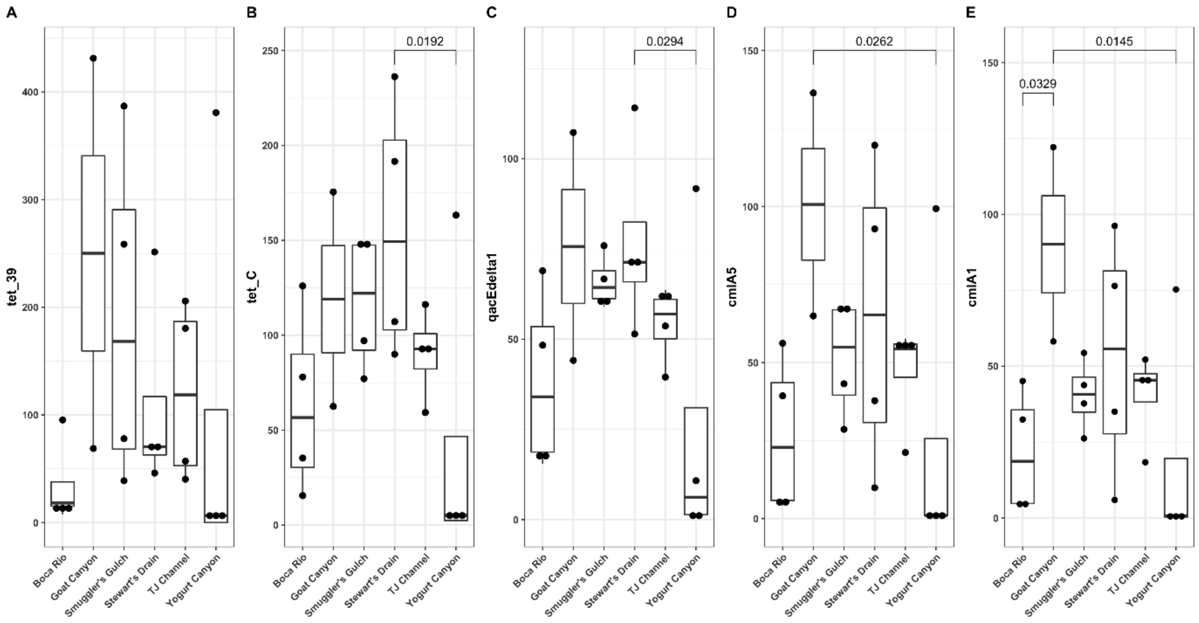

Figure S2. Genes conferring antibiotic resistance through efflux mechanisms, found at each site in the Tijuana River and Estuary.

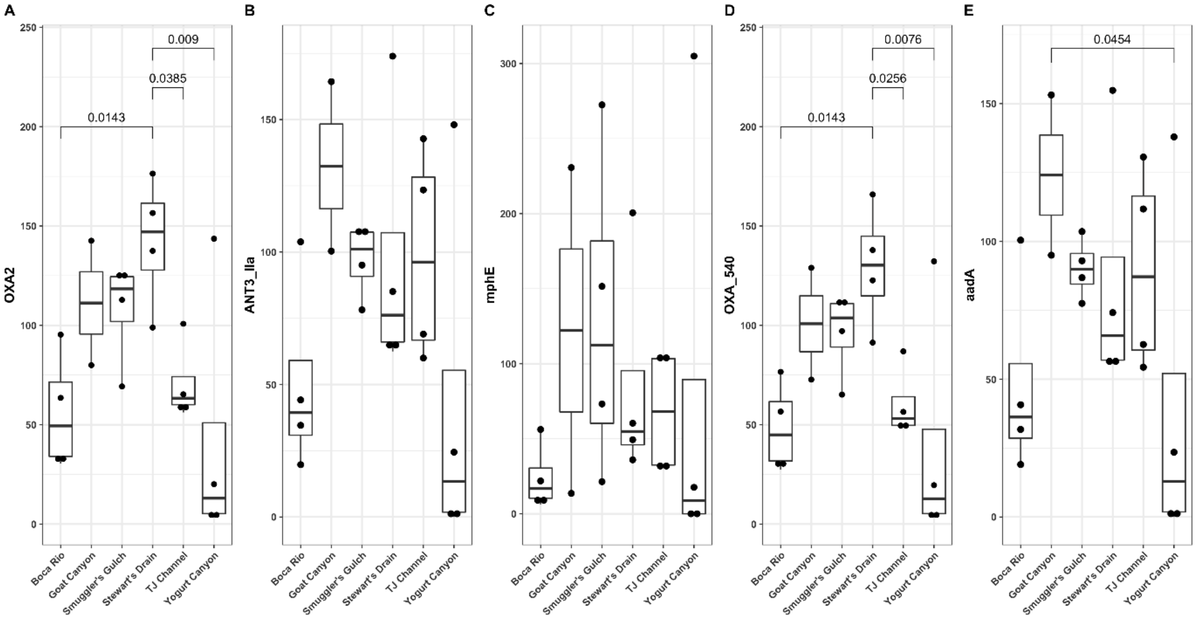

Figure S3. Genes conferring antibiotic resistance through inactivation (including beta-lactamase) mechanisms, found at each site in the Tijuana River and Estuary.

Table S1. Site names and coordinates.

Site Description	Coordinates
Boca Rio	32.559363, -117.127699
Stewart’s Drain	32.541284, -117.061214
Tijuana River Channel	32.542138, -117.038440
Smuggler’s Gulch	32.541995, -117.087917
Goat Canyon	32.542138, -117.106267
Yogurt Canyon	32.535473, -117.122625

Table S2. Sample Filtration Volumes

Sample ID	Site	Date	Amount Filtered (mL)
1	Stewart’s Drain	02/12/20	40
2	Yogurt Canyon	02/12/20	110
3	TJR Concrete Channel	02/12/20	70
4	Smuggler’s Gulch	02/12/20	70
5	Boca Rio	02/12/20	60
6	Smuggler’s Gulch	11/22/19	60
7	TJR Concrete Channel	11/22/19	80
8	Stewart’s Drain	11/22/19	100
9	Goat Canyon	11/22/19	60
10	Boca Rio	11/22/19	120
11	Yogurt Canyon	11/22/19	1000
12	Smuggler’s Gulch	12/06/19	110
13	TJR Concrete Channel	12/06/19	60
14	Stewart’s Drain	12/06/19	90
15	Goat Canyon	12/06/19	100
16	Boca Rio	12/06/19	60
17	Yogurt Canyon	12/06/19	90
18	Smuggler’s Gulch	02/24/20	120
19	TJR Concrete Channel	02/24/20	70
20	Stewart’s Drain	02/24/20	60
21	Boca Rio	02/24/20	190
22	Yogurt Canyon	02/24/20	140

Table S3. Summary table of Total Coliform and *E. coli* counts (MPN/100ml) at sampling sites between October 2019 and February 2020.

	Site					
Date	Boca Rio	Goat Canyon	Smuggler's Gulch	Stewart's Drain	Tijuana River Channel	Yogurt Canyon
Total Coliform (MPN/100 ml)						
11/22/2019	2.613E+06	2.420E+07	2.420E+07	1.011E+07	5.475E+06	3.873E+04
12/6/2019	1.785E+06	7.120E+07	4.611E+07	2.410E+07	1.050E+07	2.420E+08
2/12/2020	1.870E+07	n/a	2.420E+07	2.420E+07	2.420E+07	6.300E+04
2/24/2020	5.940E+05	n/a	3.270E+07	6.488E+08	9.090E+07	1.890E+05
E. Coli (MPN/100 ml)						
11/22/2019	6.830E+05	2.420E+07	2.420E+07	8.704E+06	4.370E+05	1.334E+03
12/6/2019	2.750E+05	1.710E+07	1.376E+07	1.483E+07	3.130E+06	6.488E+07
2/12/2020	3.448E+06	n/a	6.867E+06	5.794E+06	4.884E+06	1.000E+04
2/24/2020	5.200E+04	n/a	2.723E+06	8.360E+07	1.850E+07	1.000E+04

Table S4. HF183 and crAssphage Correlation Results

Statistical Test	*p* value
ANOVA	6.66e-10
Spearman's Correlation	3.143e-06
Kendall's Correlation	2.991e-08
Pearson's Correlation	6.659e-10
Regression Analysis	6.66e-10
